# Depth-resolved X-ray residual stress analysis on samples with cylindrically shaped surface topography

**DOI:** 10.1107/S1600576726007302

**Published:** 2026-08-24

**Authors:** Manuela Klaus, Daniel Apel, Mirko Boin, Robert Wimpory, Christoph Genzel

**Affiliations:** a Department of Microstructure and Residual Stress Analysis, Helmholtz-Zentrum Berlin für Materialien und Energie GmbH, Albert-Einstein-Straße 15, Berlin 12489, Germany; Montanuniversität Leoben, Austria

**Keywords:** X-ray residual stress analysis, curved surfaces, angle-dispersive diffraction, energy-dispersive diffraction, stress depth gradients

## Abstract

Angle- and energy-dispersive diffraction are applied to investigate the influence of the X-ray beam diameter on the results of residual stress analysis on a sample with an inhomogeneous surface topography. For sin^2^ψ measurements on parts of the surface featuring a cylindrical topography, modifications of the fundamental equation of X-ray stress analysis are proposed to correct the experimentally determined residual stresses for the rotational effect caused by the different orientation of the local principal stress coordinate system within the irradiated part of the sample. It is shown that the hoop and longitudinal stress components require different treatments in this respect. For the hoop stress component a correction factor is proposed which considers the variation in the illuminated surface area during the ψ tilt of the sample. Correction of the longitudinal stress component requires knowledge of the hoop stress component, which affects the slope of the sin^2^ψ regression line and thus partially acts as a normal stress component. For cases in which ψ tilting is not possible for geometric reasons, the applicability of the transverse contraction method is discussed. Experimental verification is carried out on a ferritic steel sample into which grooves of the same diameter were milled to different depths, resulting in different central angles.

## Abstract 2

Modifications of the fundamental equation of X-ray stress analysis are proposed that allow correction of the hoop and longitudinal stresses obtained from sin^2^ψ measurements on samples featuring an inhomogeneous surface topography. The formalism is verified by angle- and energy-dispersive measurements performed on a ferritic steel sample with cylindrically shaped regions with different central angles.

## 1. Introduction

The shape, width and position of X-ray diffraction lines depend on many factors that can be attributed to the diffractometer setup [instrumental broadening, see *e.g.* Alexander (1948 ▸, 1950 ▸), Eastabrook (1952 ▸) and Wilson (1965 ▸)] and to the (micro)structure of the investigated material itself [size and strain broadening, see *e.g.* Mittemeijer & Scardi (2004 ▸)]. Since X-ray stress analysis (XSA) is based on the precise measurement of a line’s position and its shift due to the material’s inherent (*i.e.* residual) stresses (Noyan & Cohen, 1987 ▸; Hauk, 1997 ▸), the surface topography of the investigated sample also plays an important role for measurements performed in reflection geometry. This is because the depth range below the surface from which the information in the diffraction signal originates is limited to a few tens to a few hundreds of micrometres, depending on the photon energy and the absorption of the material. A series of studies have shown that even flat surfaces, in combination with beam divergence and asymmetric scattering geometries, give rise to line shifts and thus to the formation of ‘ghost stresses’ (Zantopulos & Jatczak, 1970 ▸; Faninger, 1976 ▸), as does a wrong sample height or beam position (Fenn & Jones, 1988 ▸; Jo & Hendricks, 1991 ▸; Convert & Miege, 1992 ▸).

Technical parts and components often have a highly in­homogeneous surface topography in the form of notches, grooves or strong local curvature (*e.g.* gear wheels, springs), which places very high demands on XSA. François *et al.* (1992 ▸) have identified four effects that cause line position shifts in such cases and must therefore be taken into account in the evaluation. The shifts originate from (i) the rotation of the local stress reference system, (ii) its translation from the diffractometer centre, (iii) absorption, and (iv) partial shadowing and/or screening of the X-ray beam. These effects have been investigated in numerous theoretical and experimental studies. Many authors examine only some of these effects, such as the translation and/or the absorption effect (Doig & Flewitt, 1978*a* ▸; Doig & Flewitt, 1978*b* ▸; Dowling *et al.*, 1988 ▸; Yu & Zhang, 1989 ▸; Berruti & Gola, 2003 ▸; Rivero & Ruud, 2008 ▸) or the rotation effect (Willemse & Naughton, 1985 ▸). A holistic theoretical approach, which includes translation and rotation effects as well as absorption and shadowing, was formulated by François *et al.* (1995 ▸) and Dionnet *et al.* (1996 ▸, 1999 ▸).

A decisive criterion for the feasibility of XSA experiments on complex-shaped components is the free accessibility of the measurement point by the incident and diffracted X-ray beam. In a previous report (Genzel *et al.*, 2021 ▸) we used the energy-dispersive (ED) diffraction method (Giessen & Gordon, 1968 ▸; Buras *et al.*, 1968 ▸) to study the residual stress state at the inner walls of narrow bore holes with a large length to diameter ratio. Since the ED method provides complete diffraction patterns at fixed and small scattering angles 2θ, it was possible to analyse the residual stresses in the near-surface region of the bore hole walls both non-destructively and with depth resolution. The above-mentioned rotation effect was taken into account by a scaling factor that depends on the ratio of the X-ray beam cross section to the bore hole diameter.

A disadvantage of the small diffraction angles used in ED diffraction is that even a weakly pronounced surface topography leads to beam shadowing, which means that the longitudinal stress component in notches and grooves, for example, is not accessible for the analysis. In such cases angle-dispersive (AD) diffraction using large Bragg angles allowing for XSA measurements performed in the Ω mode (Faninger, 1976 ▸) has to be applied (Doig & Flewitt, 1978*a* ▸). However, the application of AD diffraction is limited to XSA experiments on open structures, *i.e.* it cannot be used for residual stress analysis on the inner walls of holes *etc.*

The present work aims at a systematic study of the rotation effect (ψ missetting; François *et al.*, 1995 ▸) by XSA using ED and AD diffraction performed on a ferritic steel sample with a well defined surface topography, as shown in Fig. 1 ▸. The rotation effect denotes the continuous change in the orientation of the principal axis system of the stress tensor along the curved specimen surface. In contrast, the magnitudes of the individual stress components are assumed to be constant within the irradiated surface. The investigated sample was part of a round robin test to determine residual stresses on material featuring an inhomogeneous surface topography. It was provided by MEng Jörg Behler from Sentenso Strahl­prozesstechnik GmbH. The sample is characterized by three grooves that have the same nominal diameter but have been milled to different depths into the flat surface. This results in locally curved areas with different central angles and thus different dimensions perpendicular to the grooves, which can be assumed to have an influence on the strength of the rotation effect.

The impact of curved surfaces on the residual stresses obtained by XSA has been discussed in various studies. François *et al.* (1995 ▸) and Dionnet *et al.* (1999 ▸) gave explicit expressions which allow quantification of the error for both the hoop and the longitudinal stress components as functions of the ratio of the X-ray beam cross section (*d*) and the radius of curvature (*r*_0_), the Bragg angle θ, and the tilt angle ψ. It was found that much stricter conditions regarding the *d*/*r*_0_ ratio must be fulfilled for the hoop stress component than for the longitudinal stress component. Based on these studies, the DIN standard (DIN EN 15305:2008/AC:2009; https://doi.org/10.31030/1425472) specifies limiting values for the *d*/*r*_0_ ratio above which corrections are required to the residual stresses determined experimentally using the sin^2^ψ method.

The sample shown in Fig. 1 ▸ enabled a series of investigations to study systematically how different degrees of curvature in the surface areas affect the results of XSA. As the grooves are open structures with free access of the X-ray beam to the measurement points, a combination of AD and ED diffraction could be used for the measurements. However, due to beam shadowing, the small scattering angles used in ED diffraction restricted the application of this mode to the analysis of the hoop stress component. On the other hand, AD diffraction with its larger scattering angles could be applied to study the longitudinal stress component as well. The following issues are addressed in the present paper:

(i) Analysis of the hoop stress component σ_ϕϕ_: sin^2^ψ measurements using both ED and AD diffraction with different X-ray beam cross sections. Introduction and application of a ‘pseudo-dynamic’ scaling factor to correct the stress with regard to the rotation effect.

(ii) Analysis of the longitudinal stress component σ_*yy*_: sin^2^ψ measurement using AD diffraction. Introduction and application of a ‘static’ correction factor which takes into account the influence of the hoop stress component.

(iii) Analysis of the in-plane stress component σ_∥_: application of the transverse contraction method (Genzel *et al.*, 2023 ▸) to determine an average stress value 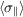 = 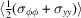 from ED diffraction measurements performed under ψ = 0°.

## 2. Treatment of the local rotation of the stress reference system in sin^2^ψ-based residual stress analysis

### 2.1. Coordinate systems: flat versus curved surfaces

XSA measurements on flat (point *A* in Fig. 1 ▸) and curved surfaces (points *B*, *C* and *D* in Fig. 1 ▸) require treatment in different reference systems with respect to the local residual stress tensors (Fig. 2 ▸). While the residual stress state in flat surface areas can be described by Cartesian coordinates (*x*, *y*, *z*), cylindrical coordinates (*r*, ϕ, *z*) should be used in areas with (strong) local curvature (Gil-Negrete & Sanchez-Beitia, 1989 ▸). This has important consequences for the normal stress component perpendicular to the surface, σ_*zz*_ in Cartesian coordinates and σ_*rr*_ in cylindrical coordinates, which is difficult to detect experimentally. Due to the boundary conditions these components must vanish at the surface, *i.e.* σ_*zz*_(*z* = 0) = 0 and σ_*rr*_(*r* = 0) = 0. Using the nomenclature of the coordinate axes in Fig. 2 ▸, the differential equilibrium conditions containing these stress components are given in Cartesian [equation (1*a*[Disp-formula fd1a])] and cylindrical coordinates [equation (1*b*[Disp-formula fd1b])] by (Timoshenko & Goodier, 1951 ▸) 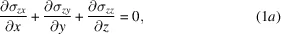

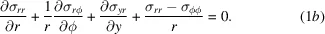
For single-phase polycrystalline materials with random texture, it follows from equation (1*a*[Disp-formula fd1a]) that σ_*zz*_ may be neglected in the evaluation, if shear stresses σ_*zx*_ or σ_*zy*_ either are not present or do at least not show lateral variations within the irradiated surface area [note that this is not valid for multiphase materials featuring a periodic structure of the phases (*e.g.* ferrite and cementite in pearlitic steel) (Hanabusa *et al.*, 1983 ▸; Ruppersberg, 1997 ▸)]. In curved surface regions, however, equation (1*b*[Disp-formula fd1b]) reads as follows in the absence of shear stress components: 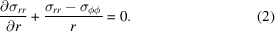
This indicates that the near-surface residual stress state for cylindrically shaped regions must be considered as multi-axial, which is due to the term connecting the radial and the hoop stress components.

### 2.2. Direction-dependent data evaluation

#### 2.2.1. Hoop stress component

In the work of Genzel *et al.* (2021 ▸) the experimentally obtained hoop stresses were corrected for the rotation effect by introducing a scaling factor sinα/α, where α is the central angle enclosing the area on the curved surface illuminated by the primary beam [see also François *et al.* (1995 ▸)]. This leads to a modification of the fundamental equation of XSA which then reads 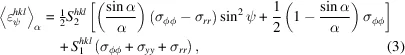
where 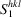 and 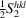 are the diffraction elastic constants. The angle brackets indicate that the lattice strain obtained for any tilt angle ψ is the mean value in the range 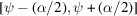. Accordingly, the stress values determined from the slope of the regression line must be multiplied by the inverse factor (sinα/α)^−1^ (hereinafter referred to as a ‘correction factor’) to obtain the true stress value.

However, this factor refers to the value of α obtained for the initial orientation of the sample, ψ = 0, and does not take into account the increase in the irradiated area (and thus in the angle α) during the ψ tilt of the sample. The situation is depicted in Fig. 3 ▸. Let *d* and *D* be the cross sections of the incident X-ray beam and the total diameter of the groove, respectively. For ψ = 0 the effectively illuminated sample area is given by 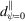 = *d*/sinθ along the groove and by 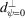 = *d* transverse to the groove. The central angle covered by the X-ray beam is 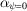 = 2arcsin(*d*/*D*), where *d* can be understood as the chord within the circular cross section of the groove.

During the ψ tilt the measurement spot elongates to *d*/(sinθcosψ) and changes its azimuthal orientation by 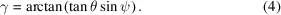
(Note that γ_max_ = θ would be reached for parallel incidence.) The extension of the irradiated area 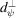 transverse to the groove (Fig. 3 ▸) then becomes 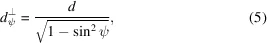
which does not depend on θ. Thus, the central angle α also becomes a function of sin^2^ψ: 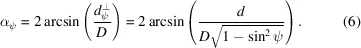
As σ_ϕϕ_ does not exist outside the groove (Fig. 2 ▸), α_ψ_ must not be greater than the maximum central angle α_max_ (Fig. 3 ▸). This boundary condition defines the maximum tilt angle ψ that can still be approached in a sin^2^ψ measurement, 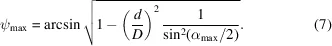
According to equations (5) and (6), the illumination transverse to the groove changes during the sin^2^ψ measurement. The same therefore also applies to the scaling factor 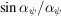 and its inverse. However, since the correction factor 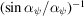 must not be applied point by point for each ψ angle, but integrally to the experimentally determined stress value [*cf*. equation (3)], the use of an averaged factor is suggested: 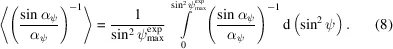
Here, 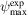 denotes the maximum ψ angle used in the measurement, for which 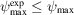 must apply [*cf*. equation (7)]. Fig. 4 ▸ reveals that the ‘static’ correction factor 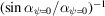 would underestimate the rotation effect.

#### 2.2.2. Longitudinal stress component

The analysis of the longitudinal stress component σ_*yy*_ needs a different treatment in order to correct the value obtained from the sin^2^ψ measurement for the rotation effect. Fig. 5 ▸ shows that beam shadowing, in contrast to the case for the hoop stress component, requires the use of large 2θ angles on the one hand and considerably restricts the accessible ψ range for the sample tilt on the other. For these two reasons, the extension of the illuminated area in the direction of the primary beam, 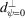 (transverse to the groove), is comparatively small and does not change significantly during the ψ tilt, *i.e.*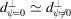 is approximately fulfilled [Fig. 5 ▸(*b*)].

However, the angular range α covered by the X-ray beam must also be taken into account for this correction, as can be seen from Fig. 5 ▸(*a*). For measurements of σ_*yy*_ in the longitudinal direction, the hoop stress component σ_ϕϕ_ acts as a transverse stress component for α = 0, and for α ≠ 0 increasingly as a normal stress component, depending on the position on the circumference of the groove. The same applies to the radial component σ_*rr*_ but in the opposite direction: for α = 0 the radial component σ_*rr*_ acts as a normal stress component, and for α ≠ 0 (again depending on the position on the circumference of the groove) it acts increasingly as a transverse stress component. The fundamental equation of XSA then takes the following form: 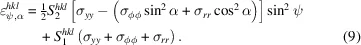
The correction of the longitudinal stress component σ_*yy*_ with regard to the rotation effect therefore requires knowledge of both the hoop component σ_ϕϕ_ and the radial stress component σ_*rr*_. In contrast to the analysis of the hoop stress component, the correction no longer appears here as a factor but is a difference term in the slope of the sin^2^ψ regression line. For the calculation of a mean correction term, it can be assumed that the surface area illuminated for each ψ (and therefore also α) remains approximately constant. Then one obtains from the subtrahend in the square bracket term of equation (10)
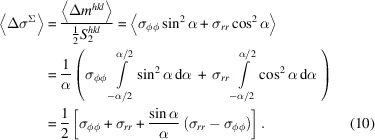
Here, 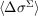 is the contribution of the two stress components σ_ϕϕ_ and σ_*rr*_ by which the component σ_*yy*_ appears reduced or enlarged (depending on its sign) in the sin^2^ψ analysis, *i.e.*
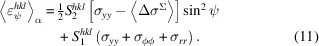
Fig. 6 ▸ depicts the situation for the different central angles α that characterize the grooves of the sample investigated in the present paper. Small (large) α correspond to small (large) irradiated surface areas within the groove. The shaded area, which is limited by the dashed and dotted curves, can be regarded as an uncertainty range that characterizes the influence of the radial stress component σ_*rr*_, which is unknown in many cases because it is difficult to detect. The diagram also shows that σ_*rr*_ either reduces or increases the contribution of the hoop stress component σ_ϕϕ_ to the difference term 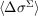 depending on its sign.

#### 2.2.3. In-plane stress analysis

The considerations in the preceding sections were based on the assumption that the geometric constraints of the surface topography allow at least a ψ tilt up to angles that enable the application of the sin^2^ψ method. For the sample investigated in the present study this condition is fulfilled [Fig. 7 ▸(*a*)], but this is no longer the case for complex-shaped technical components such as gears or very deep channels, as shown in Fig. 7 ▸(*b*). Under certain conditions, however, information about the residual stress state can still be obtained. For materials with cubic crystal symmetry and pronounced elastic anisotropy defined by the Zener factor *A* = 2*c*_44_/(*c*_11_ − *c*_12_) (Zener, 1948 ▸; Chung & Buessem, 1967 ▸) (*c*_*ij*_ are the single-crystal constants), the transverse contraction method introduced by Genzel *et al.* (2023 ▸) can be applied. In addition to a large Zener factor (for ferritic steel, *A* = 2.4), this method assumes the absence of composition gradients and that the residual stress state in the depth range covered by the X-ray beam is homogeneous and biaxial.

The concept of the transverse contraction method is shown in Fig. 8 ▸. It consists of the evaluation of differences 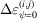 = 
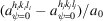
 of lattice strains that were obtained for a series of *N* reflections *hkl* in the diffraction pattern obtained for ψ = 0.

Here and in the following, *a*^*hkl*^ = 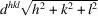 stands for the *d* spacings normalized to the edge length of the unit cell *a*^100^, and *a*_0_ is the strain-free lattice parameter. This procedure allows for the evaluation of ½*N*(*N* − 1) lattice strain differences (Genzel *et al.*, 2023 ▸), 
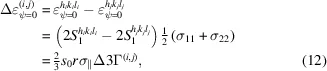
where *s*_0_ = 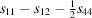 (*s*_*ij*_ are single-crystal elastic moduli) and *r* denotes the weighting factor between the Reuss model and the Voigt grain interaction model. This leads to 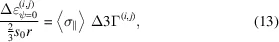
which is a linear equation *y* = *mx* whose slope corresponds to the average in-plane residual stress 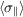 = 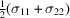 for Cartesian and 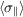 = 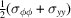 for cylindrical coordinates. Note that the transverse contraction method described above is based on the evaluation of strain differences and is therefore almost insensitive to uncertainties in the strain-free lattice parameter *a*_0_. In this respect, the transverse contraction method differs from strain-scanning techniques that use monochromatic synchrotron or neutron radiation (Webster *et al.*, 1996 ▸; Withers & Webster, 2001 ▸). In these cases, the lattice strain is usually determined for only one reflection, which means that even small uncertainties in *a*_0_ lead to large error margins in the residual stresses.

On the other hand, the weighting factor *r*, which describes the grain-interaction model and thus the degree of anisotropy of the material, has a significant influence. It can be determined using an optimization method (Klaus & Genzel, 2019 ▸; Genzel *et al.*, 2023 ▸), the application of which in the present case resulted in a value of *r* ≃ 0.4, which corresponds to the Eshelby–Kröner model (Eshelby, 1957 ▸; Kröner, 1958 ▸).

## 3. Experimental

### 3.1. Sample material

As already indicated in the *Introduction*[Sec sec1], the sample studied here was part of a round robin investigation to determine residual stresses on curved surfaces. The sample is a quenched and tempered ferritic steel 42CrMo4, which was subsequently annealed at 400°C with a target hardness value of about 45–48 HRC. The sample was vacuum hardened and then shot peened with the aim of generating the highest possible residual stresses while keeping the roughness low.

### 3.2. X-ray residual stress analysis

#### 3.2.1. Diffraction

XSA measurements were performed in the AD diffraction mode on the five-circle diffractometer ETA (Genzel, 2001 ▸) and in the ED diffraction mode using the eight-circle diffractometer LEDDI (Apel *et al.*, 2018*a* ▸; Apel *et al.*, 2018*b* ▸). The parameters used for the measurement are compiled in Table 1 ▸. The measurements in the ED diffraction mode were carried out with a two-detector setup in which both detectors were arranged in a horizontal scattering geometry (Genzel *et al.*, 2025 ▸).

Fig. 9 ▸ shows the dimensions of the measurement spot irradiated by the primary beam on the sample for the initial case ψ = 0°. Since the incidence angle for the ED diffraction mode is significantly smaller than that for the AD diffraction mode, the white primary beam on the sample has a comparable longitudinal extension to the monochromatic beam, although its cross section is only about a quarter of the latter.

To determine the maximum ψ range for analysing the hoop stress, it must be ensured that the irradiated surface area does not exceed the groove during the ψ tilt. Fig. 10 ▸ demonstrates the situation for both the AD and ED cases. Comparison of Figs. 10 ▸(*a*) and 10 ▸(*b*) reveals why a correction of the rotation effect is only necessary for measurements performed in the AD diffraction mode. The reason for this is that the angle γ, which describes the azimuthal rotation of the surface area irradiated on the sample at ψ tilt according to equation (4), is significantly smaller in the ED case than in the AD case, even for large ψ values (chosen here with ψ_max_ = 71.5°). This means that only a comparatively small area (defined by α_max_) is illuminated in the grooves, resulting in a negligible (within the scope of the measurement uncertainties) averaged correction factor of 1.02. On the other hand, for measurements in the AD diffraction mode, γ takes on comparatively large values even at small ψ angles due to the significantly larger Bragg angles. The largest possible tilt angle ψ_max_, for which the irradiated surface is just within the groove, is thus determined by the central angle of the groove according to equation (7).

#### 3.2.2. Data evaluation

The procedure for evaluating the residual stress depth profiles from the sin^2^ψ measurements will be explained using the example of measurement point *A* (flat surface) which serves as a reference. Here, as in all other measurement points and directions, no ψ splitting was observed, which indicates the absence of shear stresses (Fig. 11 ▸). The classical sin^2^ψ method (Macherauch & Müller, 1961 ▸) was used for residual stress analysis, *i.e.* the stress values were determined from the slope *m*^*hkl*^ of the regression lines fitted to the sin^2^ψ distributions, which proved to be linear in all cases. The strain-free lattice parameter *a*_0_ required to determine the residual stress via σ = 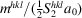 was taken from the intersection of the regression line with the strain-free direction of a biaxial residual stress state, which is given by 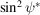 = 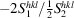 (Fig. 11 ▸).

The discrete residual stress depth profiles 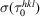 in Laplace space were obtained by plotting the individual stress values against the maximum information depth (Klaus & Genzel, 2019 ▸), which is given by 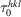 = 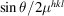 (ED case) or 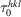 = 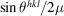 (AD case), where μ is the linear absorption co­efficient [Fig. 12 ▸(*a*)]. In the second step use was made of the transformation 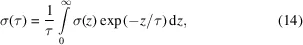
which relates the experimentally accessible stresses in Laplace space to the stresses in real or *z* space. The following approach was used to describe the residual stress depth profiles in real space: 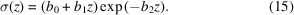
The Laplace transform of the above expression is obtained by applying equation (14),
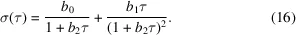
The unknown parameters were determined by least-squares fitting of equation (16) to the experimentally obtained discrete depth profiles 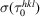 [Fig. 12 ▸(*b*)]. The differences at the ordinate axes of the residual stress depth profiles indicate that the sin^2^ψ method cannot be used to separate the in-plane stress component to be determined from the out-of-plane component perpendicular to the surface [σ_*zz*_ and σ_*rr*_ in the case of flat (measurement point *A*) or curved surfaces (measurement points *B*–*D*)]. The analysis of the residual stress component σ_*yy*_ (not shown here) resulted in an almost identical depth profile, from which a rotationally symmetrical in-plane residual stress state can be concluded.

## 4. Results

### 4.1. Hoop stress

According to the considerations in Section 3.2.1, it can be assumed for the analysis of the hoop stress component in the grooves that the depth profiles determined by ED diffraction do not require any correction with regard to the rotation effect. However, this does not apply to the analyses performed in the AD diffraction mode, as the beam cross section was significantly larger for these measurements (Fig. 9 ▸). As can be seen from Fig. 10 ▸(*c*), the residual stress values determined from the slope of the regression lines must be corrected with average correction factors 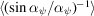, which vary depending on the size of the central angle of the individual grooves.

Fig. 13 ▸ indicates that the application of the correction factors shown in Fig. 10 ▸(*c*) leads to a significant shift of the stresses determined by AD diffraction towards higher compressive stresses, which then fit into the Laplace stress depth profiles determined by ED diffraction. The quantitative differences between the three measurement points can be seen in the enlarged sections in Fig. 14 ▸. Obviously, there is a direct correlation between the shape of the grooves and the respective correction factor required to correct the experimentally determined residual stress values.

Finally, Fig. 15 ▸ reveals, with the example of measurement point *B*, that the ‘static’ correction factor 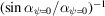 introduced by Genzel *et al.* (2021 ▸) would underestimate the influence of the rotation effect in the present case, while the use of the averaged factor 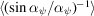 provides realistic results.

### 4.2. Longitudinal stress

Due to the unfavourable geometry, the analysis of the longitudinal residual stress component σ_*yy*_ in the grooves required the use of large Bragg angles and thus the application of the AD diffraction method. With the 220 reflection (2θ^220^ = 124.4°; Table 1 ▸) ψ tilting was possible up to ψ = 45° before beam shadowing prevented further tilting of the sample. The results in Fig. 16 ▸ show that the uncorrected and corrected residual stress values are almost identical for all measurement points, regardless of the central angle of the respective groove.

This is because the surface area illuminated by the X-ray beam in the grooves only corresponds to a mean α value of 67° (almost independent of the ψ tilt, *cf*. Fig. 5 ▸), which is thus smaller than the smallest central angle (90° for measurement point *D*). According to equation (10) and neglecting the radial stress component σ_*rr*_ (see discussion in Section 5), this yields an average correction term 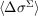 = 0.107σ_ϕϕ_. For σ_ϕϕ_ in the correction term, those values were used that correspond to the same depth 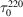 = 10.4 µm to which the values for the σ_*yy*_ component are assigned. It is also noticeable that the corrected values for the longitudinal residual stress component practically coincide with the depth profiles for the hoop stress component σ_ϕϕ_ at the corresponding depth. From this, a rotationally symmetrical in-plane residual stress state can be concluded, which thus provides the precondition for the applicability of the transverse contraction method shown in the next section.

### 4.3. Average in-plane stress

The residual stress depth profiles determined in the preceding sections from the sin^2^ψ data sets open up the possibility of verifying the applicability of the transverse contraction method outlined in Section 2.2.3. The corresponding results obtained for measurement point *B* are summarized in Fig. 17 ▸. Fig. 17 ▸(*a*) indicates that the normalized lattice spacings obtained for all reflections except 110 are arranged on the ordinate sin^2^ψ = 0 as demanded by the elastic anisotropy of the material (*i.e.* the larger 3Γ^*hkl*^ is, the smaller the lattice strain and spacings; *cf*. Fig. 8 ▸). However, this relationship only applies to such reflections *hkl* for which the sin^2^ψ method provides an approximately homogeneous residual stress level [Fig. 17 ▸(*c*)].

In the example considered here, this holds true for the diffraction lines located in the green shaded area at approximately −900 MPa. The analysis of the 110 reflection closest to the surface yields a significantly smaller residual stress value of approximately −700 MPa, which results in a smaller slope of the 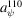
–sin^2^ψ distribution (not shown here) and thus also of the ordinate intercept 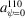 [red shaded areas in Figs. 17 ▸(*a*) and 17 ▸(*c*)]. The precondition for the applicability of the transverse contraction method (homogeneous stress state) is therefore not fulfilled, so that the 110 reflection must not be included in the evaluation. The application of the transverse contraction method to the other reflections results in an average in-plane residual stress value 〈σ_∥_〉 that fits into the σ_ϕϕ_ residual stress level of the depth range covered by them. Since the method is based on data derived from reflections *hkl* to which different information depths 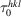 are assigned, the result obtained for 〈σ_∥_〉 can be assigned to an average depth 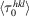, which in this case is approximately 21 µm.

## 5. Discussion and conclusions

The ferritic steel sample analysed in this study is characterized by a structured surface topography and treatment to generate a well defined residual stress state in the surface layer. The grooves, milled to varying depths, enabled systematic investigation of the influence of various beam geometry effects on X-ray stress analysis. The focus of the present paper was on the examination of the rotation effect, which includes the local variation in the orientation of the stress reference coordinate system in (strongly) curved surface areas in the evaluation. While the influence of other effects, such as deviation from the centre of the diffractometer (translation effect) and partial beam shadowing, can be verified (and corrected if necessary) by measuring stress-free powder at the same measurement point, this does not apply to the rotation effect.

It has been shown that the corrections required for both the hoop stress component σ_ϕϕ_ and the longitudinal stress component σ_*yy*_ depend in different ways on the area irradiated in the grooves, and thus on the ratio of the beam cross section *d* to the groove diameter *D*. For the hoop stress component σ_ϕϕ_, it is mandatory additionally to take into account the increase in the irradiated area during the ψ tilt of the sample. Fig. 18 ▸ shows how the results obtained in this study can be classified in relation to the recommendations given in the literature. The DIN standard suggests that ‘the irradiated area should be smaller than 0.4 times the radius of curvature of the analysed surface in the direction of the stress component to be determined’ (DIN EN 15305:2008/AC:2009; https://doi.org/10.31030/1425472), which would correspond to the case shown in the middle of Fig. 18 ▸. Applying the formalism in Section 2.2.1 to this ratio *d*/*D* = 0.2 yields a relative error for the hoop stress component of 8%. More detailed rules for limiting the relative error to values smaller than 10%, which take into account the respective diffraction conditions (Bragg angle and maximum tilt angle), are provided by François *et al.* (1995 ▸) and Dionnet *et al.* (1999 ▸). For the scenarios investigated in this paper, Fig. 18 ▸ reveals that the measurements performed using ED diffraction on the LEDDI diffractometer do not require any corrections within the scope of measurement accuracy and can therefore serve as a reference for the AD measurements on the ETA diffractometer.

Knowledge of the longitudinal stress component σ_*yy*_ is not necessary for correcting the hoop stress component σ_ϕϕ_. However, this does not apply to the radial component σ_*rr*_, which cannot be neglected *a priori* in the evaluation according to equation (2), as is often done for the σ_*zz*_ component in XSA measurements on flat surfaces. Since σ_*rr*_ must be zero directly at the surface, it can only occur as a gradient, the steepness of which depends on various parameters such as the manufacturing process, the material’s microstructure and the surface treatment. Therefore, the occurrence of the σ_*rr*_ component within the rather small information depth accessible by means of X-ray diffraction must be considered separately for each specific case. Since the sin^2^ψ method only provides the difference between the respective in-plane stress component and the out-of-plane stress component, this was taken into account in the relevant diagrams.

The correction for the rotational effect of the longitudinal stress component σ_*yy*_ requires knowledge of the hoop stress component σ_ϕϕ_ and (if present in the accessible depth range) of the radial component σ_*rr*_. According to equation (9), the two components act proportionally and in opposite directions as out-of-plane stress components and therefore lower the actual longitudinal stress component by a certain amount. Due to beam shadowing, the analysis of σ_*yy*_ in Ψ mode is only possible using AD diffraction at large Bragg angles and only up to rather small ψ tilt angles. According to Fig. 5 ▸(*b*), the requirements for the *d*/*D* ratio necessary for error minimization in the case of the longitudinal stress component are therefore less stringent than those for the hoop stress component. For all three measurement points *B*, *C* and *D*, the contribution of the hoop stress component is approximately 11%, regardless of the central angle of the groove (Fig. 16 ▸), *i.e.* following equation (11) the slope of the regression line is composed of the difference (σ_*yy*_ − 0.11σ_ϕϕ_).

Table 2 ▸ summarizes important criteria that must be taken into account when analysing the two residual stress components considered here.

The application of the transverse contraction method outlined in Section 2.2.3 is subject to strict preconditions, such as cubic crystal symmetry, pronounced elastic anisotropy, and negligible residual stress and composition depth gradients. Therefore, it should only be used if the sin^2^ψ-based methods fail for geometric reasons as depicted in Fig. 7 ▸(*b*). For the sample studied in this paper, these geometric constraints did not occur, providing an opportunity to test the above criteria. While the first two criteria for ferritic steel are already met, the homogeneity of the near-surface residual stress state in particular requires case-specific verification. The example shown in Fig. 17 ▸ demonstrates that a residual stress gradient leads to a violation of the order in which the normalized lattice spacings determined under ψ = 0 must occur according to their elastic deformability defined by the orientation factor 3Γ^*hkl*^. This finding can be used to assess whether and to what extent the transverse contraction method may be applied. In the present case of a steep stress gradient near the surface, a robust mean value for the in-plane residual stress could be determined by omitting the first reflection 110, which clearly deviates from the above order.

Finally, note that it is difficult to make a general estimate of the extent to which the uncertainties of the correction factors introduced in this work contribute to the uncertainties that generally affect X-ray measurements of residual stresses. In addition to the inevitable scattering of experimental data, these uncertainties primarily include systematic uncertainties that relate, for example, to the grain interaction model used to calculate the diffraction elastic constant, the influence of texture or the underestimation of plastic deformation. However, it can be assumed that the better the conditions for their applicability are met, the more accurately the correction factors will shift the experimentally determined residual stress values toward the actual values. The most important of these conditions is the lateral homogeneity of the residual stress state in the irradiated surface region. Within certain limits, this can already be achieved by the experimental setup itself, by keeping the cross section of the X-ray beam as small as is reasonable from the viewpoint of intensity and grain statistics.

## Figures and Tables

**Figure 1:**
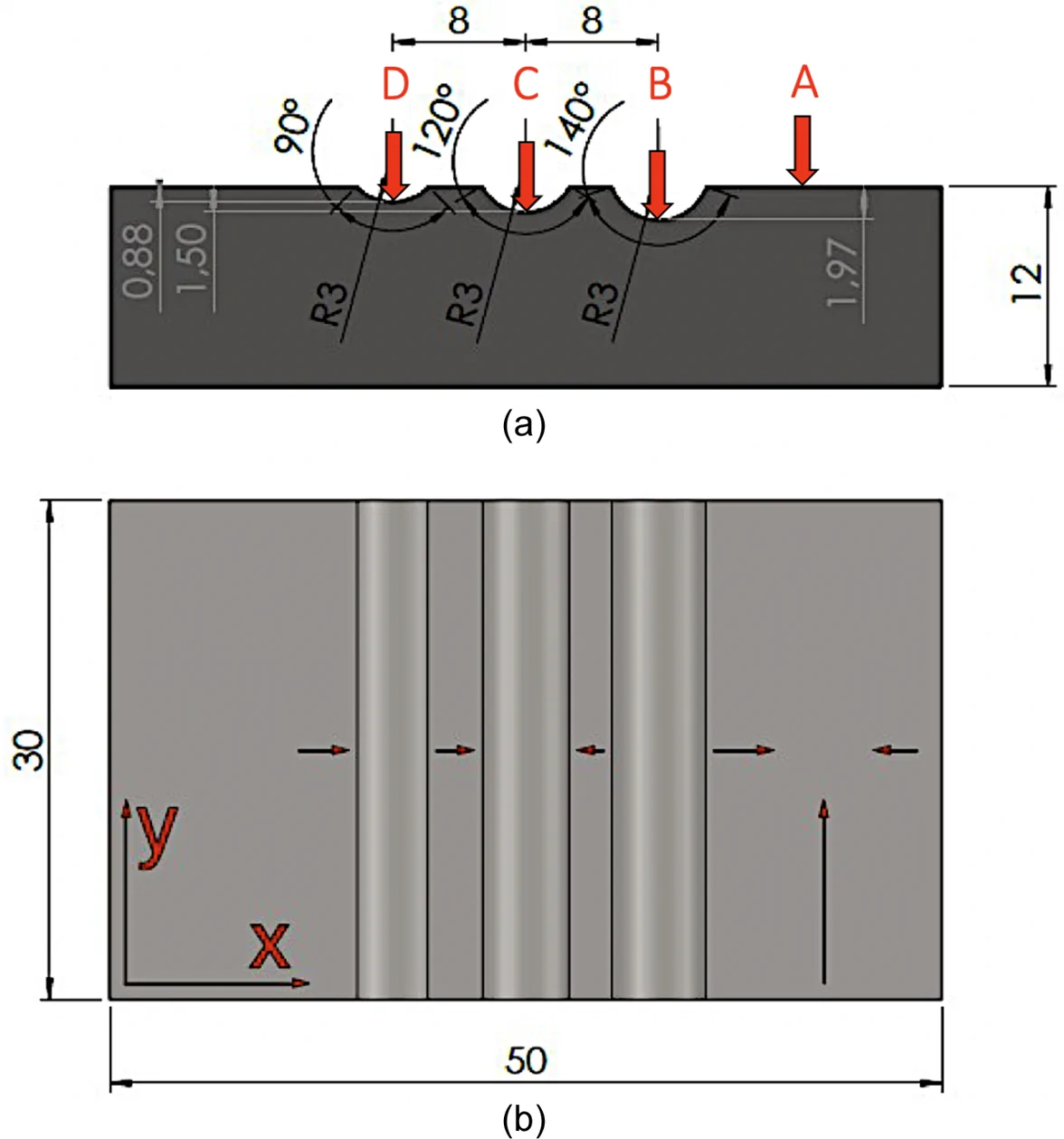
Schematic view of the investigated sample. (*a*) Front view and (*b*) top view. The arrows mark the measurement points. Diagram courtesy of the initiator of the round robin test, MEng J. Behler.

**Figure 2:**
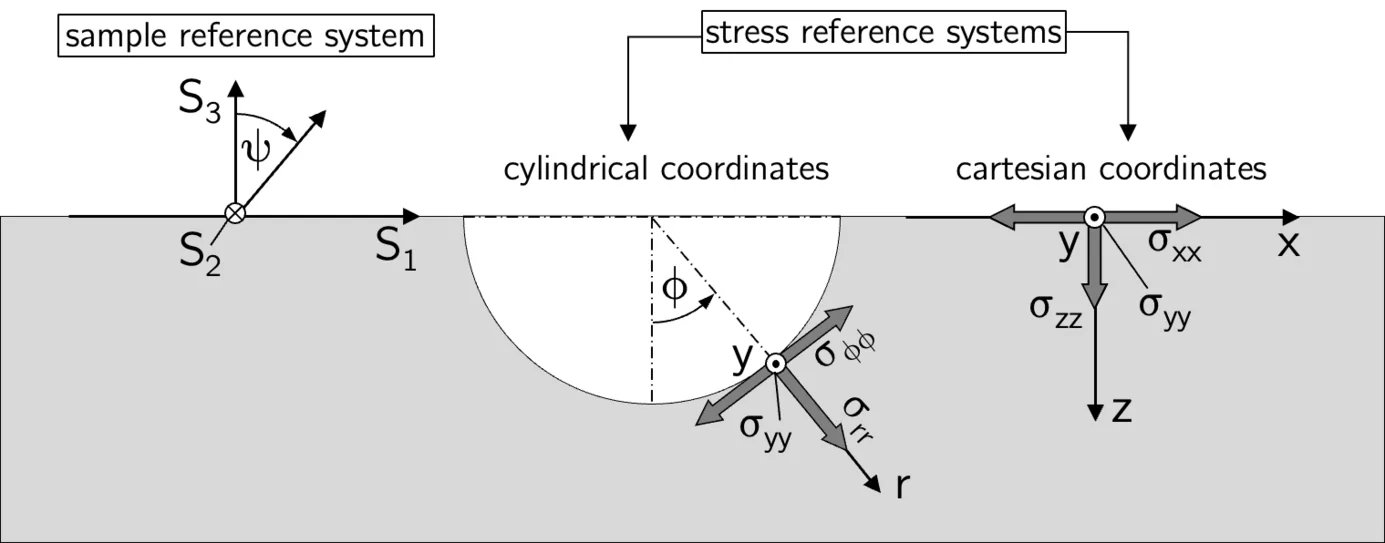
Reference systems for XSA on flat and curved surfaces. All coordinate systems are right handed. In the reference systems of the sample (stresses), the *y* axis points into (out of) the drawing plane. For the sake of clarity, only the normal components are shown in the stress reference systems.

**Figure 3:**
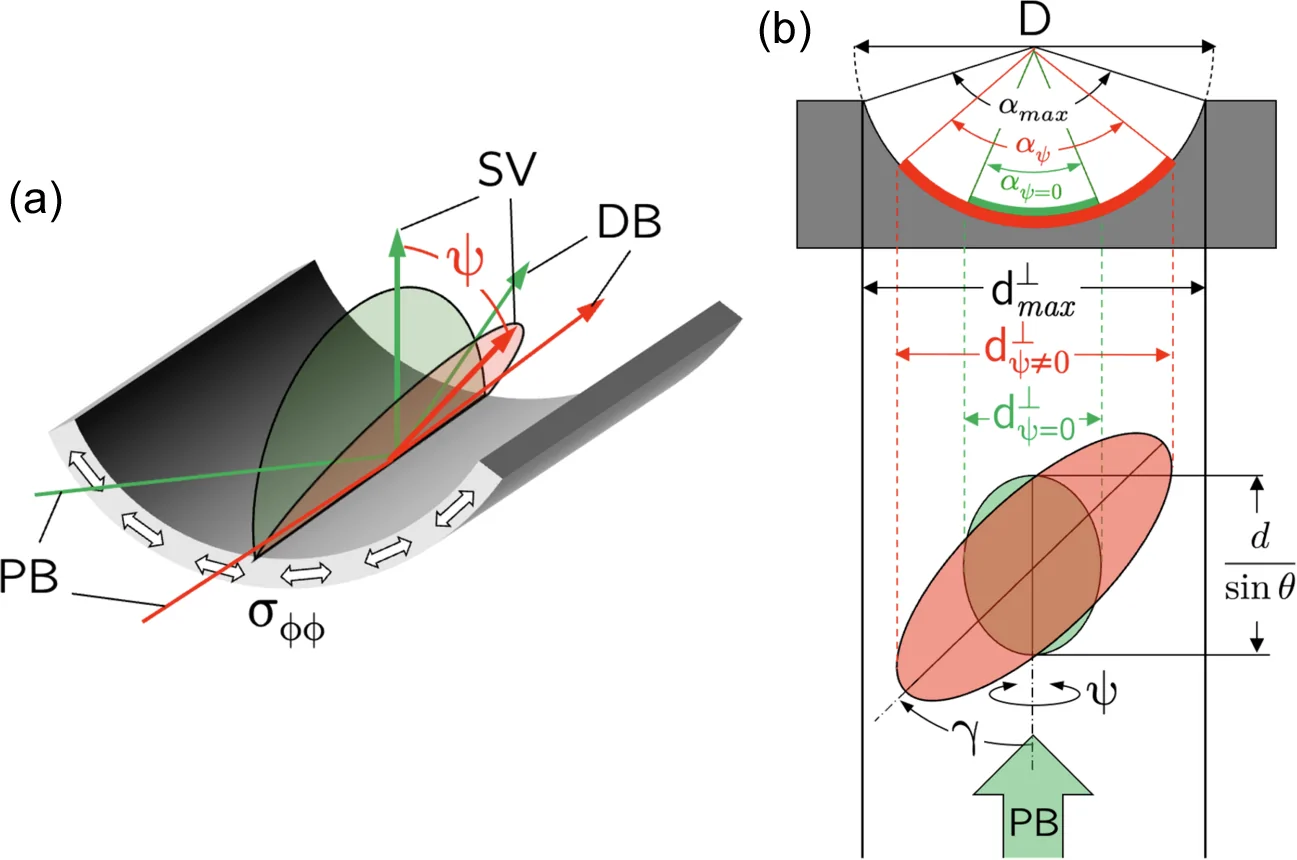
Evolution of the irradiated surface area during hoop stress analysis. (*a*) Orientation of the diffraction plane within the groove for the initial (green) and tilted (red) sample positions. (*b*) The corresponding illuminated surface areas. PB, DB and SV denote the primary beam, the diffracted beam and the scattering vector, respectively.

**Figure 4:**
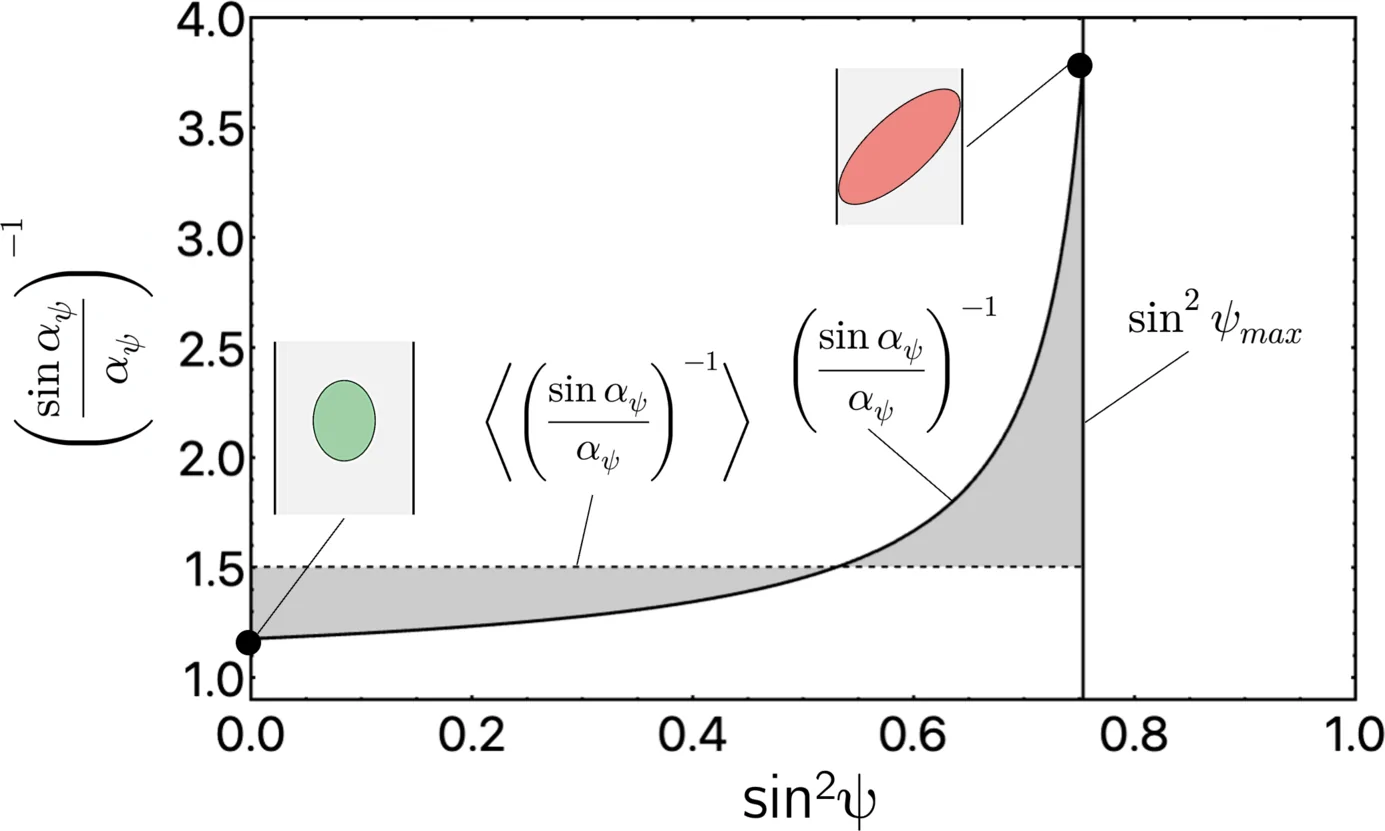
Correction factor and its average according to equations (6) to (8), calculated for *d* = 2.8 mm and *D* = 6 mm, values corresponding to the AD XSA experiments in the present paper. The curves were plotted up to the maximum tilt angle ψ_max_ = 60.2° accessible for a central angle α_max_ = 140° (measurement point *B* in Fig. 1).

**Figure 5:**
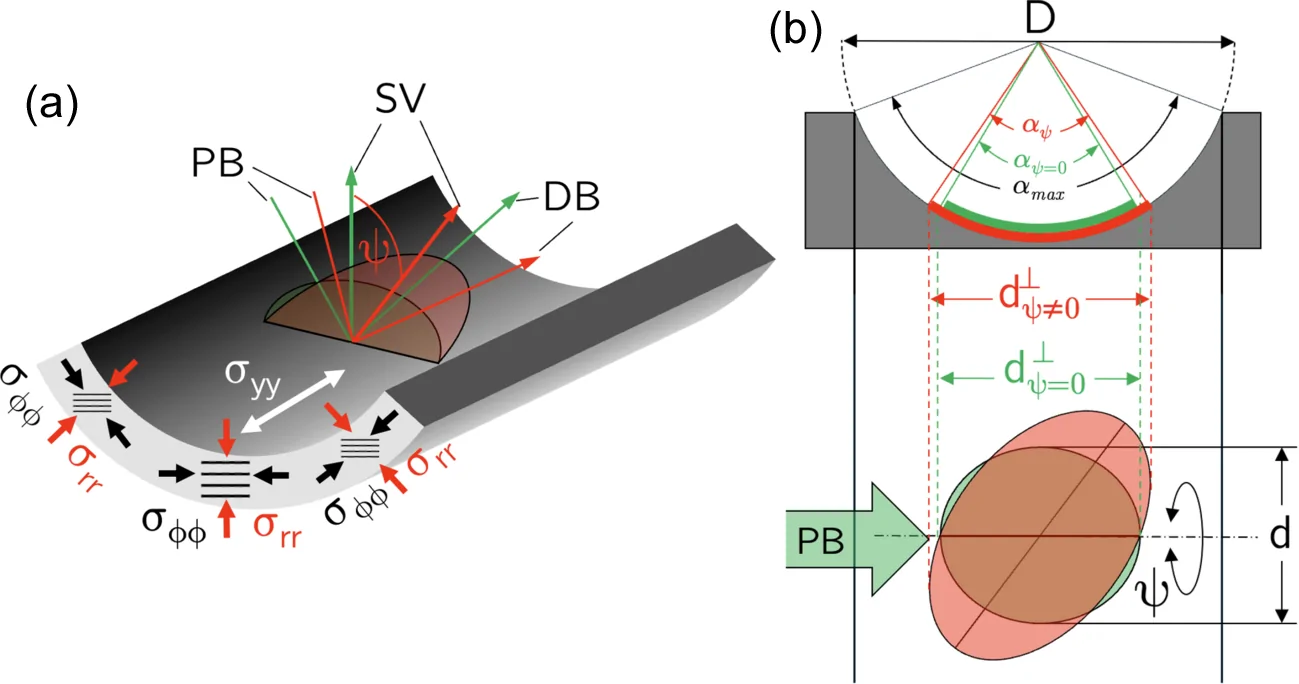
Diffraction geometry for analysis of the longitudinal stress component σ_*yy*_. (*a*) Spatial illustration of the beam path and the impact of the stress components σ_ϕϕ_ and σ_*rr*_. (*b*) True-to-scale representation of the irradiated surface area in the groove for AD diffraction at the 220 reflection (2θ^220^ = 124.4°; Table 1). For the nomenclature, see Fig. 3.

**Figure 6:**
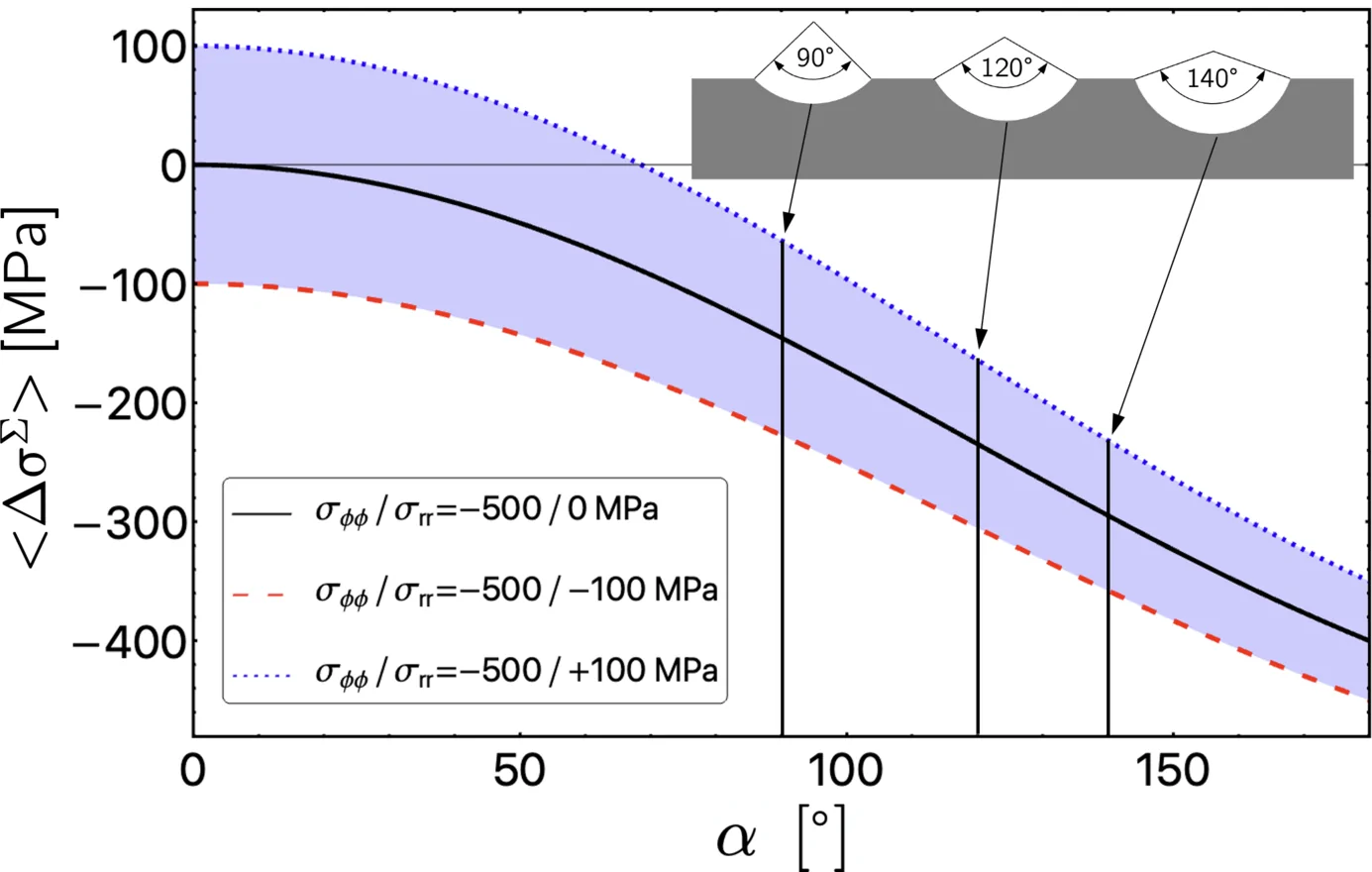
Impact of the stress components σ_ϕϕ_ and σ_*rr*_ on XSA of the longitudinal stress component σ_*yy*_ according to equations (10) and (11) (simulation). The black solid line represents the case of negligible radial stress σ_*rr*_ in the accessible information depth. The dashed red line and the dotted blue line indicate the influence of certain amounts of σ_*rr*_ featuring opposite signs.

**Figure 7:**
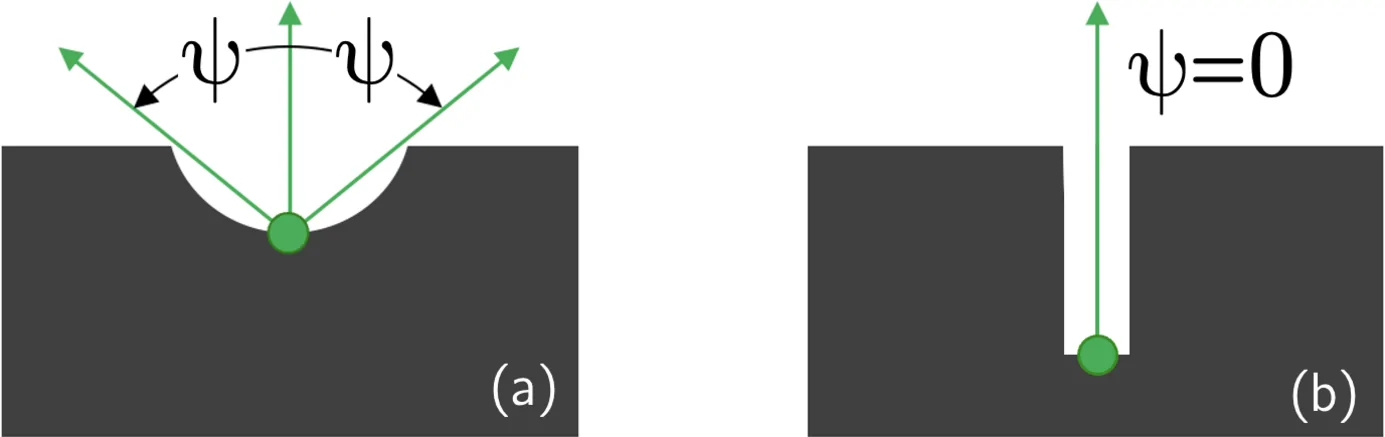
Different shapes of complex surface topography. (*a*) Shallow groove, allowing application of the sin^2^ψ method. (*b*) Deep channel, ψ tilt not possible.

**Figure 8:**
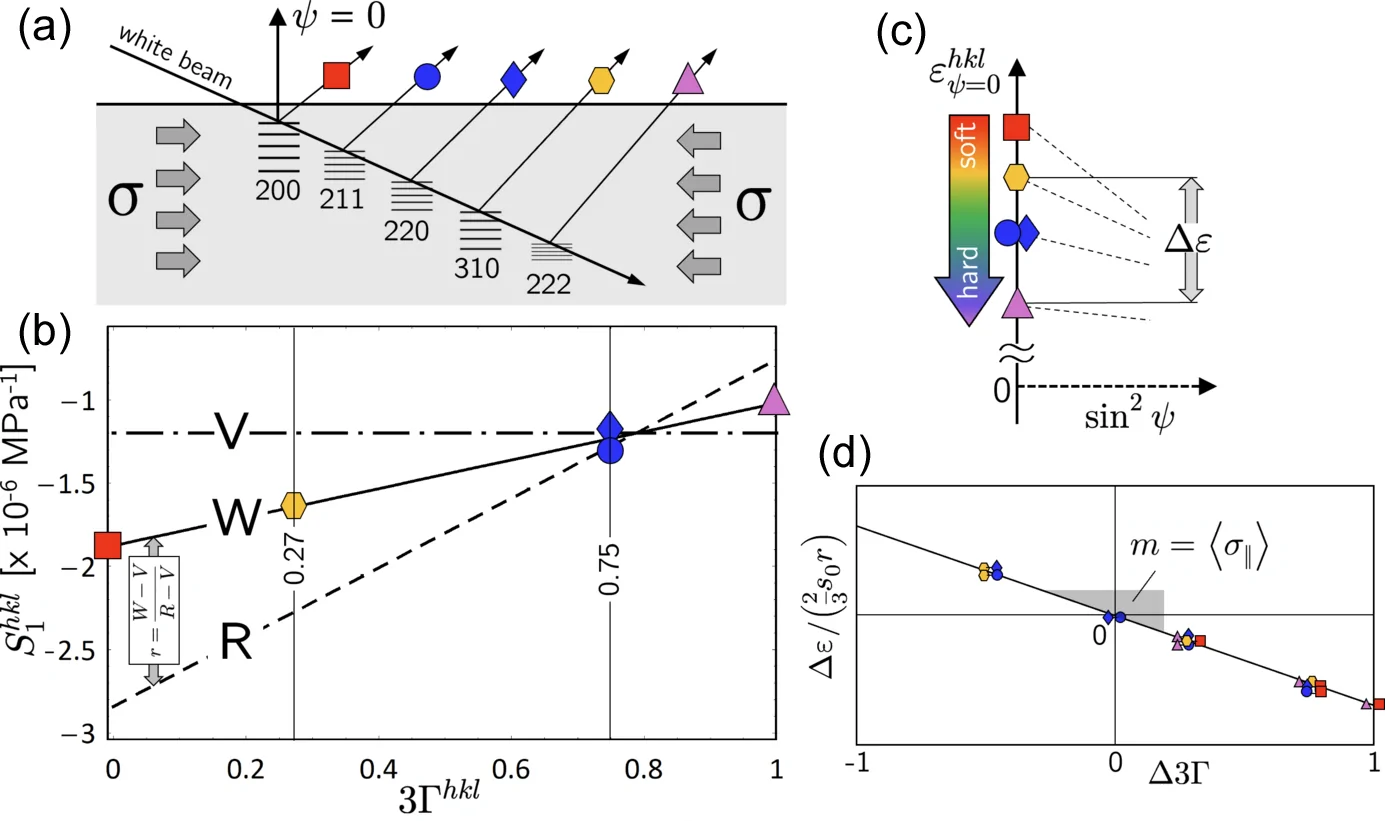
The transverse contraction method. (*a*) ED diffraction under ψ = 0. (*b*) Diffraction elastic constant 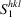 of ferritic steel as a function of the orientation factor 3Γ^*hkl*^ = 3(*h*^2^*k*^2^ + *k*^2^*l*^2^ + *l*^2^*h*^2^)/(*h*^2^ + *k*^2^ + *l*^2^)^2^ including the reflections *hkl* marked in panel (*a*). V, R and W stand for, respectively, the grain interaction models of Voigt (homogeneous strain; Voigt, 1910 ▸) and Reuss (homogeneous stress; Reuss, 1929 ▸) and a model that weights between Voigt and Reuss. (*c*) Position of the lattice strains 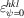 on the ordinate axis of the sin^2^ψ diagram. (*d*) Plot of the lattice strain differences (indicated by squares, triangles *etc*.) versus Δ3Γ according to equation (13). For further details see the main text.

**Figure 9:**
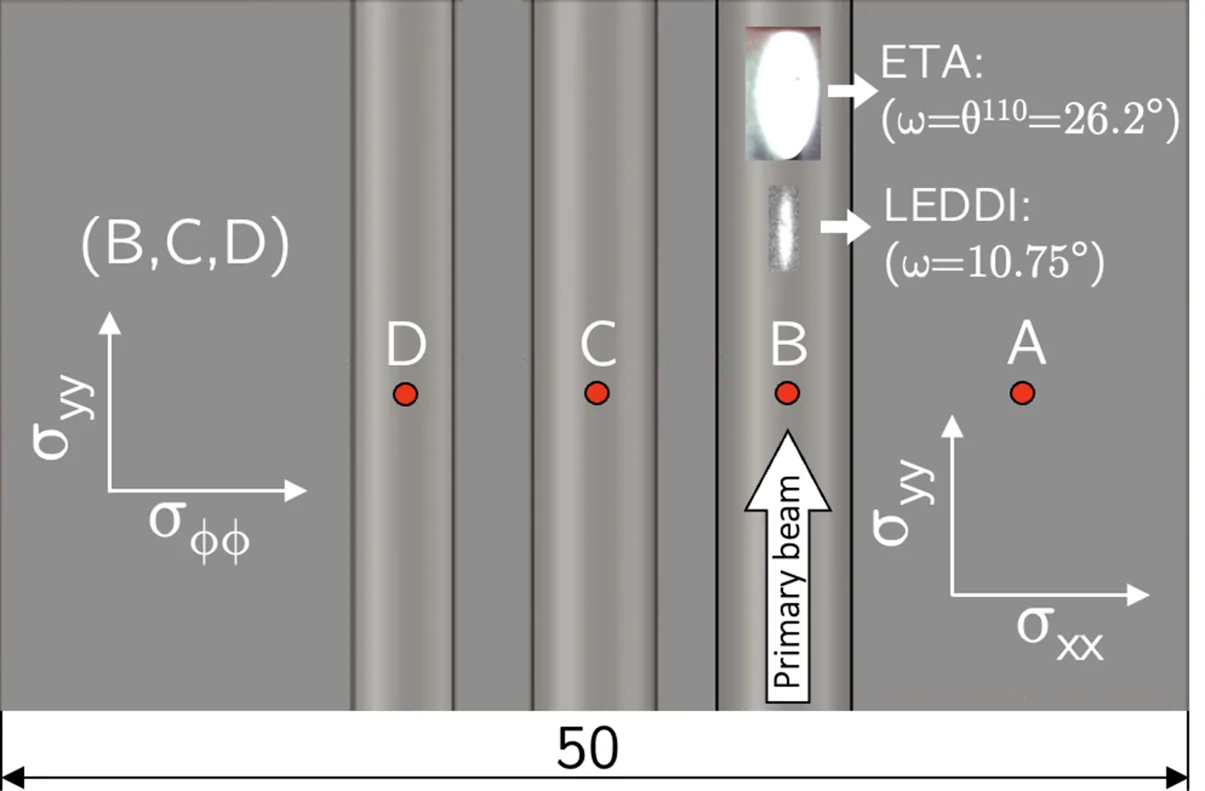
X-ray beam spot sizes on the sample (recorded on a fluorescent screen, inserted true to scale) for the initial orientation ψ = 0°. For the AD diffraction mode, the case of the 110 reflection with the smallest Bragg angle is shown, as it leads to the largest extension of the beam spot on the sample.

**Figure 10:**
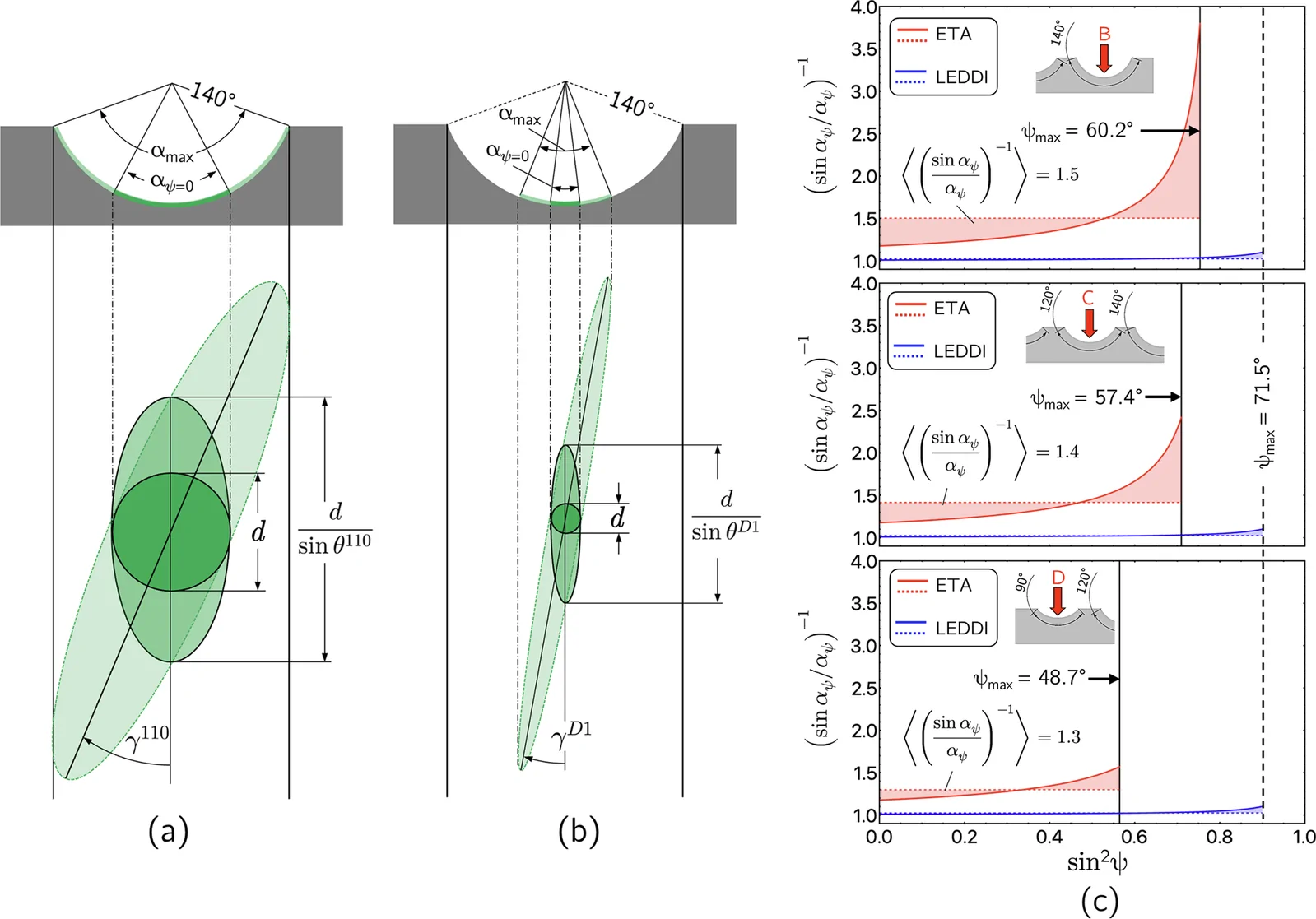
Evolution of the irradiated surface area within the groove during ψ tilting for the analysis of the hoop stress component σ_ϕϕ_, showing true-to-scale representation for measurement point *B* (groove with central angle α = 140°). (*a*) AD and (*b*) ED diffraction mode. *d* denotes the respective primary beam cross section. (*c*) ψ-dependent correction factors (sin α_ψ_/α_ψ_)^−1^ with α_ψ_ according to equation (6) (solid lines) and averaged correction factors 〈(sin α_ψ_/α_ψ_)^−1^〉 according to equation (8) (dashed lines) for measurement points *B*, *C* and *D*, respectively. For further details see the main text.

**Figure 11:**
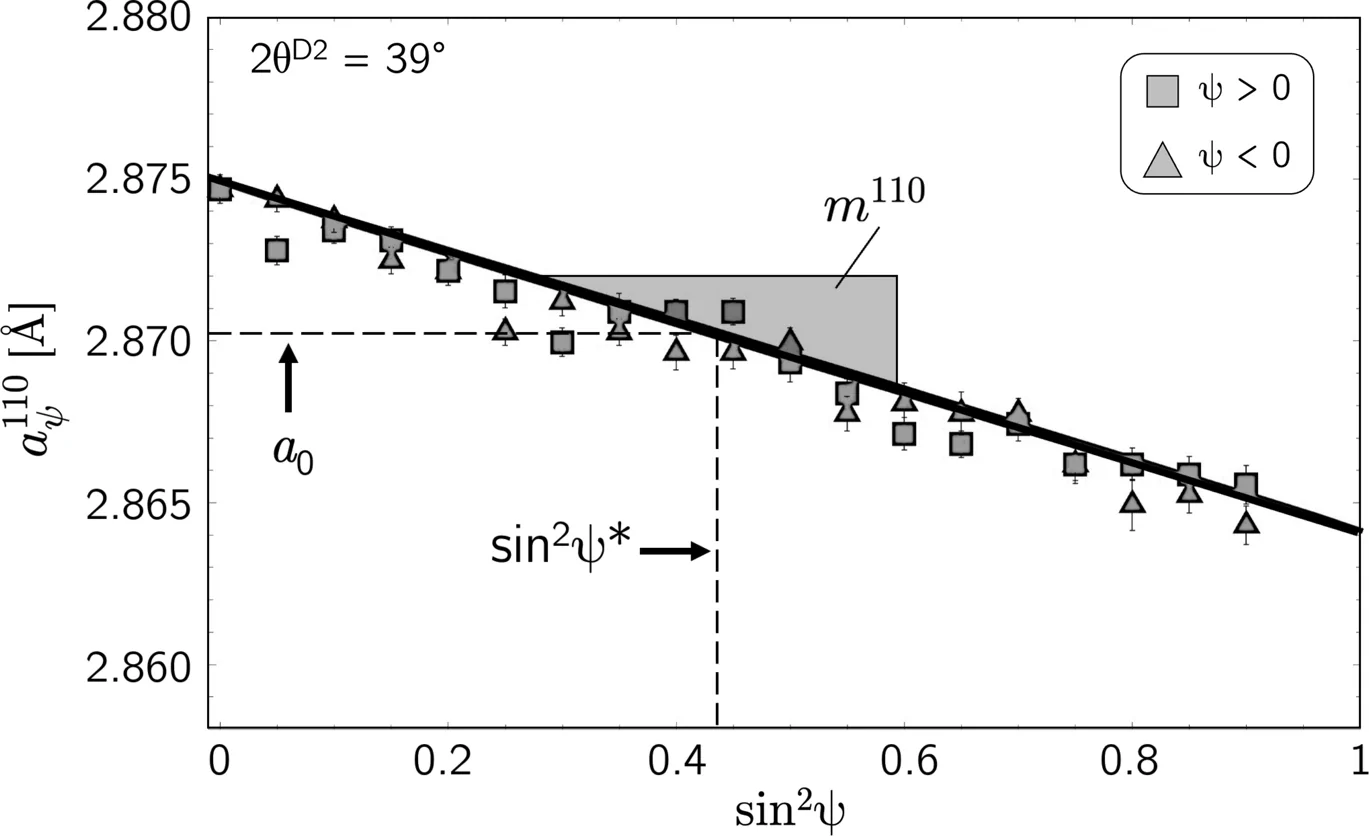
ED XSA carried out at point *A* in the *x* direction (*cf*. Fig. 1). 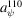
–sin^2^ψ distribution obtained with detector D2. The maximum tilt angle ψ_max_ for all measurements performed in the ED diffraction mode was chosen as 71.5°. For the AD measurements ψ_max_ was calculated according to equation (7) [*cf*. Fig. 10(*c*)].

**Figure 12:**
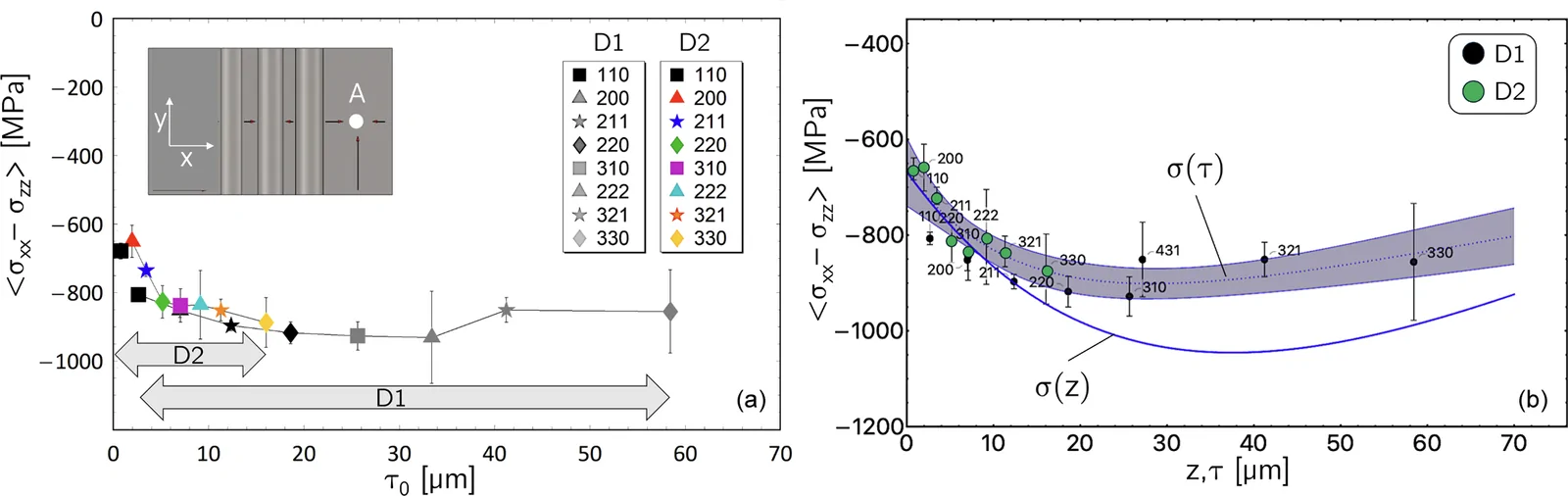
Residual stress depth distribution from the sin^2^ψ measurement in the ED diffraction mode carried out in the *x* direction at point *A*. (*a*) Laplace stress depth profile σ(τ). The double arrows indicate the depth ranges covered by the two detectors D1 and D2. (*b*) Determination of the real-space stress depth profile σ(*z*) by inverse Laplace transform [*cf*. equations (14) to (16)]. The shaded areas mark the confidence intervals in Laplace space which comprise 95% of the discrete data points.

**Figure 13:**
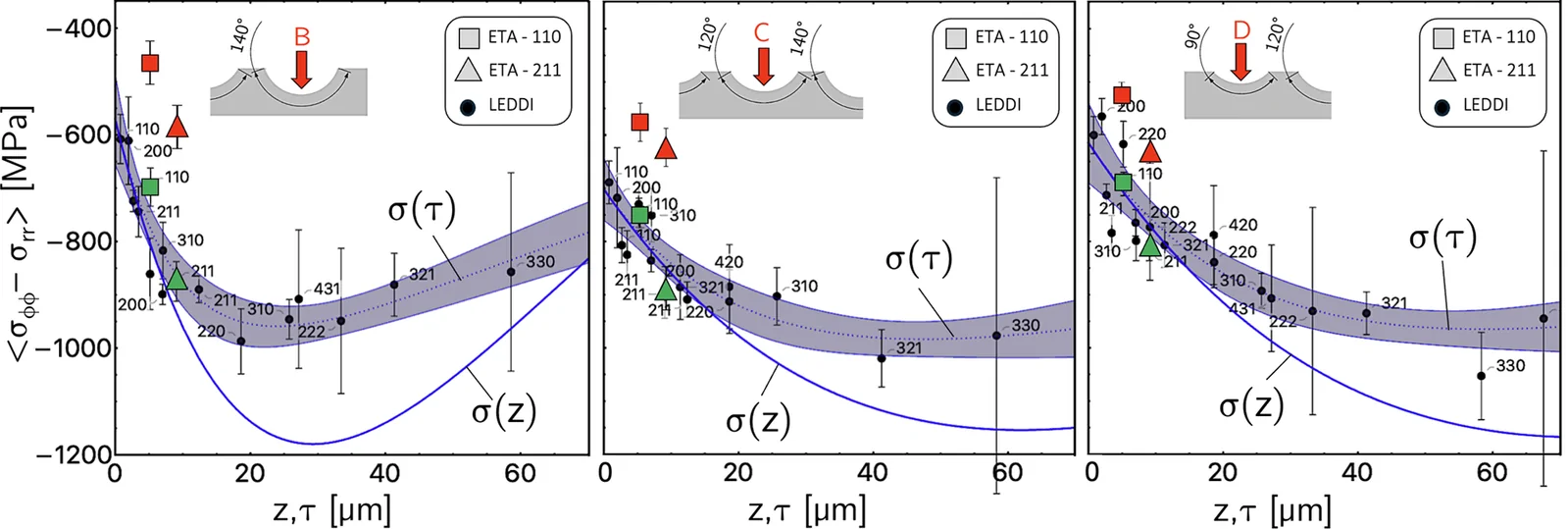
Hoop stress depth profiles determined in the grooves. The black dots mark the values obtained by ED diffraction (not divided into detectors D1 and D2 for reasons of clarity). The red (green) symbols indicate the results of AD diffraction without (with) correction of the rotation effect described in Section 2.2.1. For the meaning of the shaded areas, see Fig. 12.

**Figure 14:**
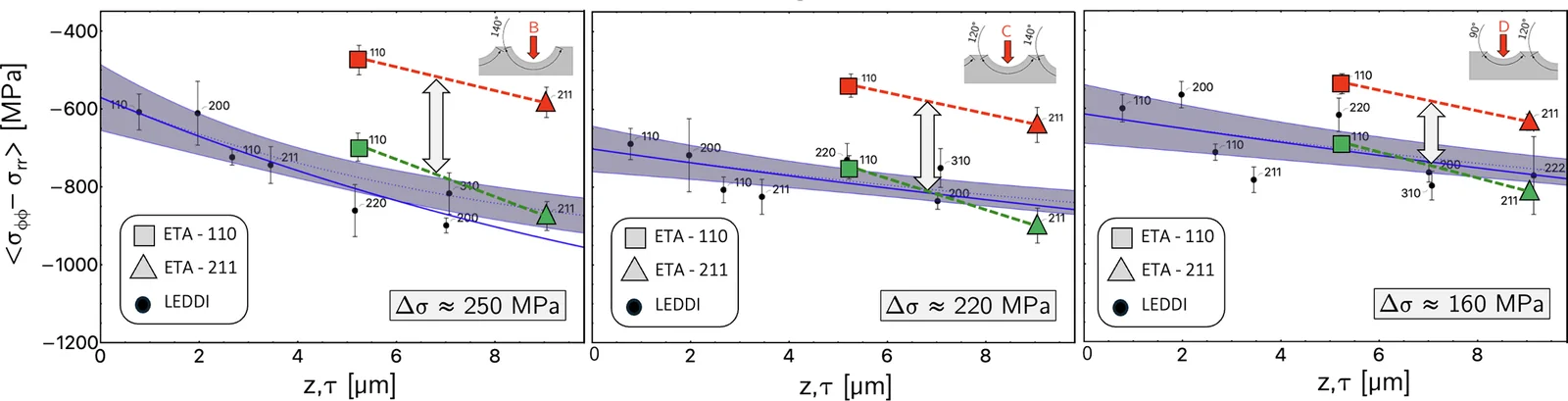
Enlarged sections of Fig. 13, showing the relationship between the depth of the grooves (described by their central angle) and the differences between the uncorrected and corrected residual stresses obtained by AD diffraction.

**Figure 15:**
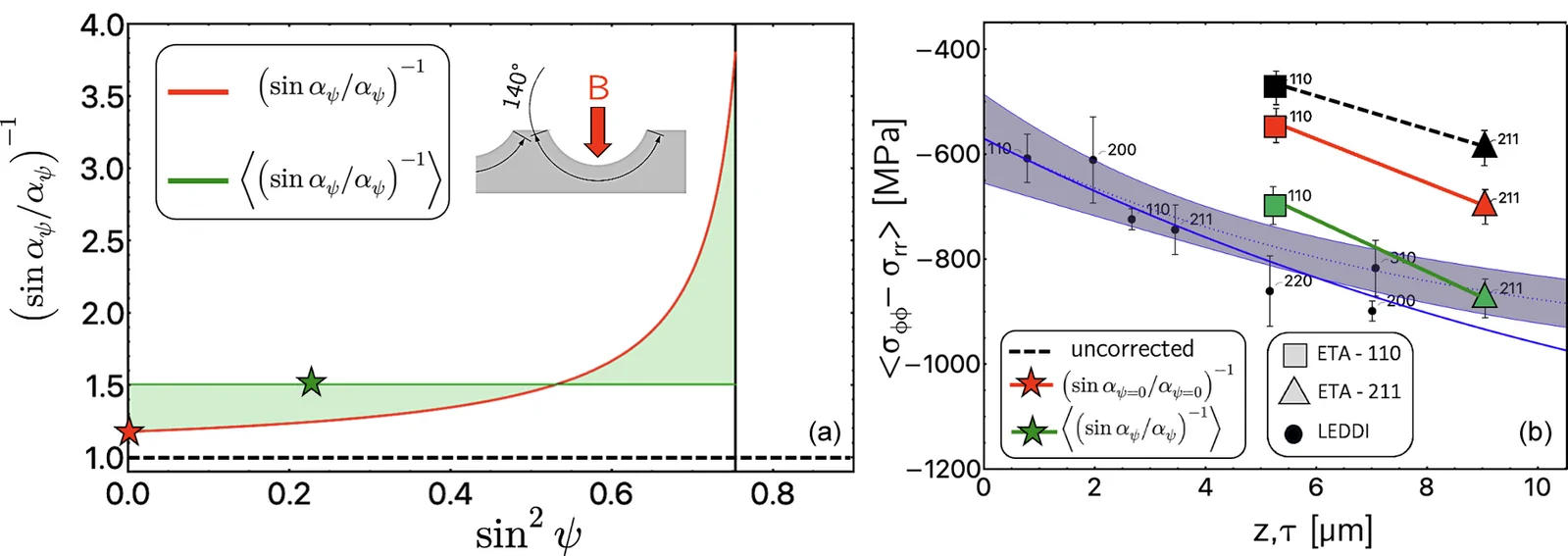
Application of the ‘static’ and averaged factors to correct the hoop stress with regard to the rotation effect. (*a*) ψ dependency of the correction factors. (*b*) Shift of the hoop stress values obtained experimentally by AD diffraction using the ‘static’ (red) and the averaged (green) correction factors, respectively.

**Figure 16:**
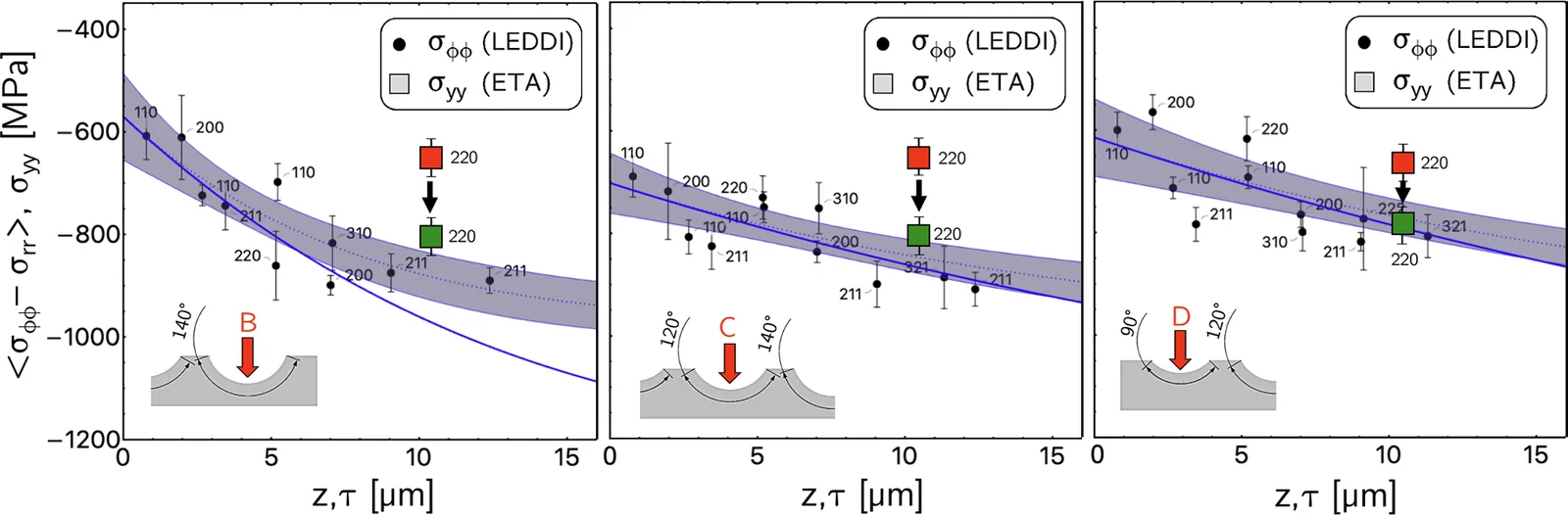
Analysis of the longitudinal stress component in the grooves. The red (green) squares indicate the uncorrected (corrected) σ_*yy*_ values. The Laplace (dotted lines) and real-space (solid lines) depth profiles of the hoop stress component σ_ϕϕ_ are included for the purpose of classification of the near-surface residual stress state.

**Figure 17:**
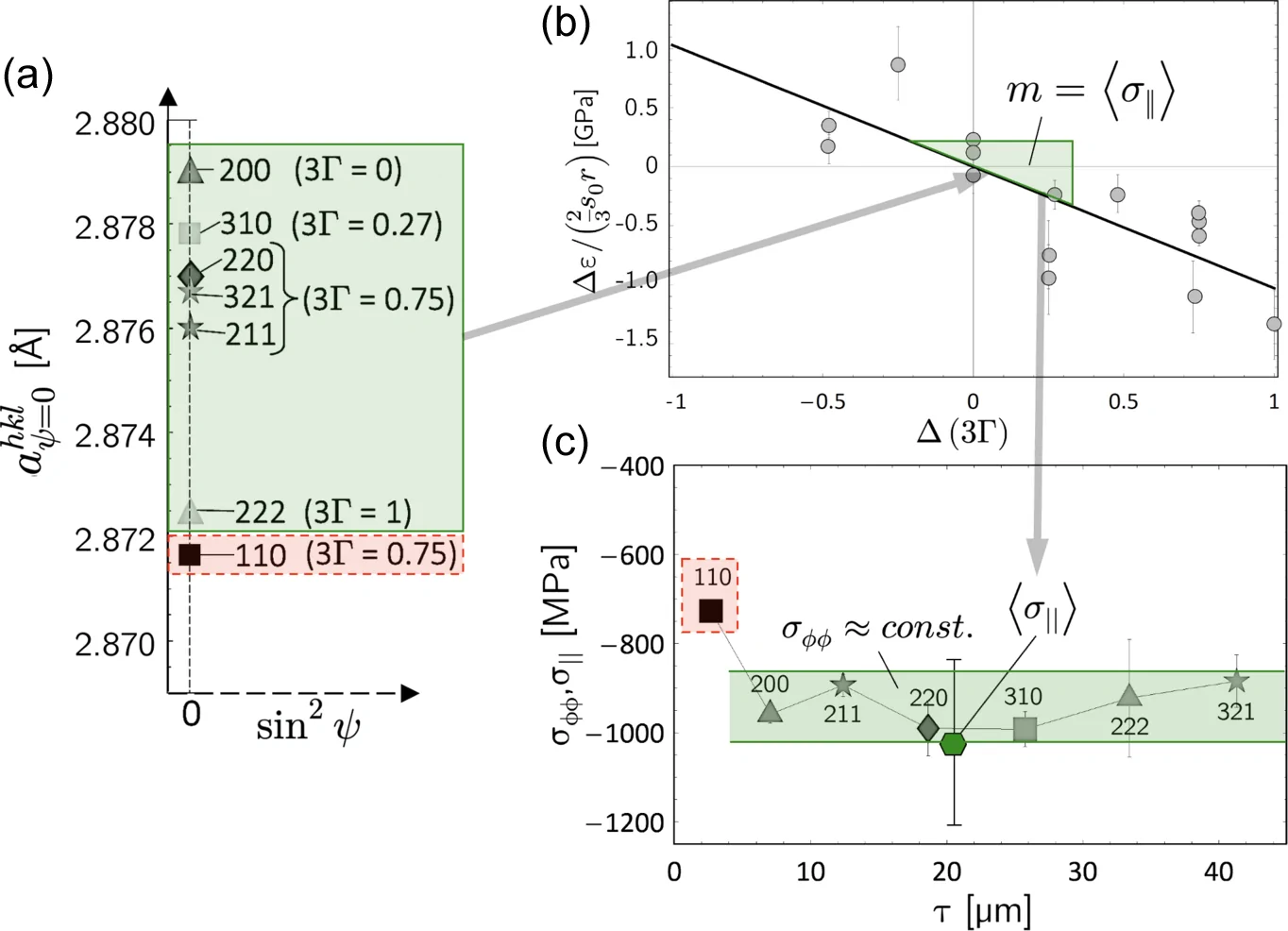
Application of the transverse contraction method to measurement point *B*. (*a*) Normalized lattice spacings obtained under ψ = 0 with detector D1 (2θ = 21.5°). (*b*) Data evaluation according to equation (13). (*c*) Discrete residual stress depth profile 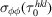 evaluated from the D1 data, and averaged in-plane residual stress 〈σ_∥_〉 obtained from the slope of the regression line fitted to the data in panel (*b*). The green shaded areas in panels (*a*) and (*c*) mark the reflections that result in an almost homogeneous residual stress depth profile. For further details see the main text.

**Figure 18:**
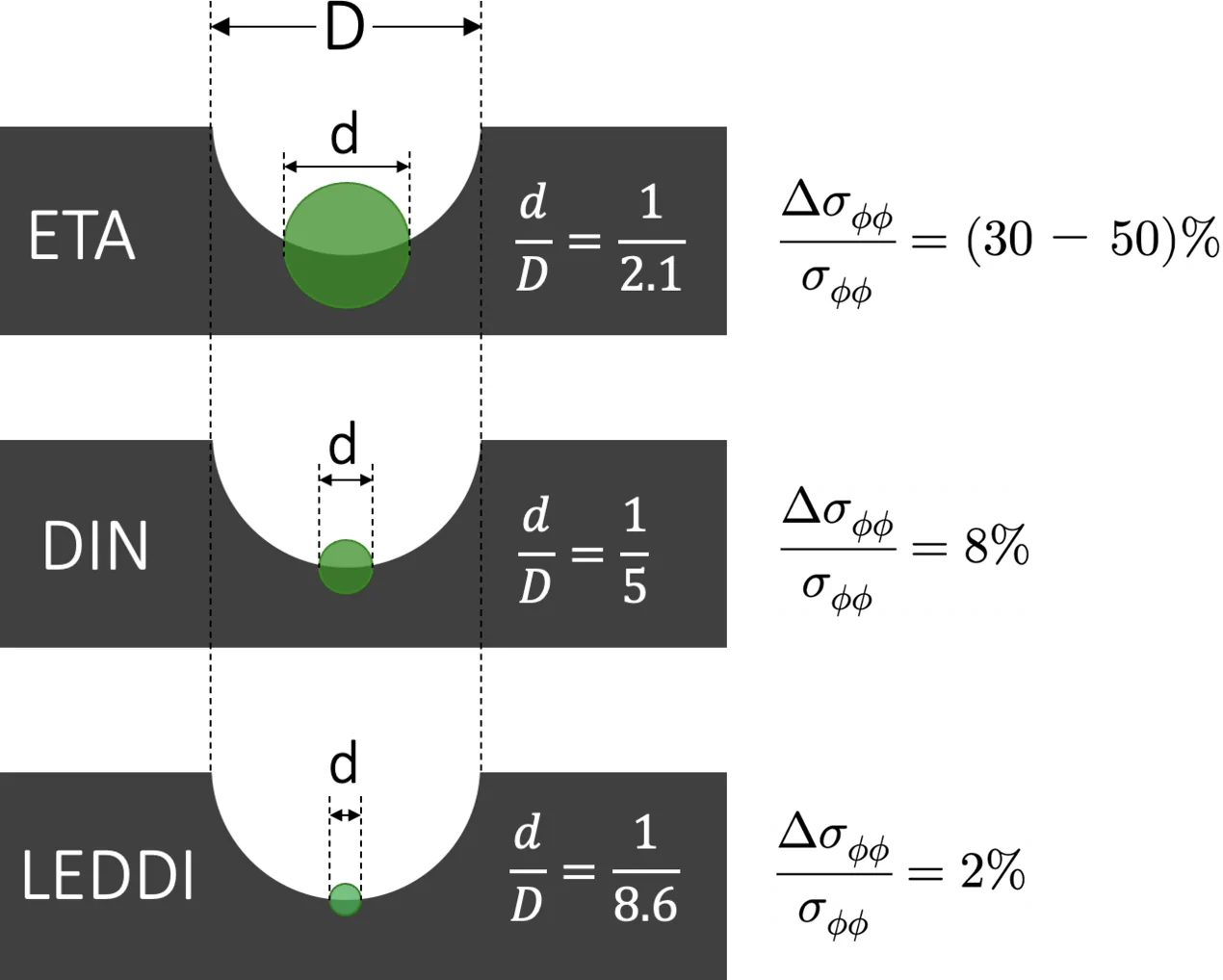
Relative errors of the hoop stress component according to equation (8) for different ratios *d*/*D*. The range specified for the AD measurements on the ETA diffractometer refers to the various central angles α_max_ = 90°–140° of the grooves *D*, *C* and *B*. DIN denotes the recommendation of the standard (DIN EN 15305:2008 ▸/AC:2009 ▸; https://dx.doi.org/10.31030/1425472). For further details see the main text.

**Table 1:** Parameters applied in the diffraction experiments

	ETA	LEDDI
Diffraction mode	Angle-dispersive	Energy-dispersive
X-ray source	Co long-fine focus tube (40 kV/45 mA)	W long-fine focus tube (60 kV/45 mA)
Detector	Scintillation counter	Ketek Si-drift detectors
Optical elements	Primary beam: polycapillary semi-lens; diffracted beam: Soller slit (FWHM = 0.4°) + 001 LiF monochromator	Primary beam: collimator (Ø = 0.5 mm); diffracted beam: equatorial Soller slits (FWHM = 0.2°)
Beam cross section at the sample	Ø = 2.8 mm	Ø = 0.7 mm
XSA mode (sin^2^ψ analysis)	Symmetrical Ψ mode, evaluated diffraction lines: 2θ^110^ = 52.4°, 2θ^211^ = 99.7°, 2θ^220^ = 124.4°	Data acquisition in Ψ mode with two detectors D1 and D2: 2θ^*D*1^ = 21.5°, 2θ^*D*2^ = 39°, ω = θ^*D*1^ = 10.75°
Integration time	10–30 s per angular step (Δ2θ = 0.05°)	4000 s per spectrum
Reference measurements	sin^2^ψ measurements on gold powder in the grooves to quantify the influence of the surface topography (translation and beam shadowing effect)	
Diffraction elastic constants	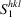 and 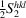 calculated using the single-crystal elastic constants taken from Landoldt–Börnstein (1984 ▸) by the Eshelby–Kröner grain-interaction model	
Stress components	σ_ϕϕ_ and σ_*yy*_	σ_ϕϕ_

**Table 2:** Parameters and factors influencing the analysis and correction of residual stress components in the hoop and longitudinal directions on curved surfaces

	Hoop stress σ_ϕϕ_	Longitudinal stress σ_*yy*_
Applicable diffraction mode	AD, ED	ED
Correction influenced by other stress components?	(σ_*rr*_)	σ_*rr*_, σ_ϕϕ_
Main impact factors	Beam cross section	Amount of the transverse component σ_ϕϕ_
	Bragg angle	Size of the central angle α

## Data Availability

The data presented in this study are available on request from the corresponding author.
